# Intelligent Cockpits for Connected Vehicles: Taxonomy, Architecture, Interaction Technologies, and Future Directions

**DOI:** 10.3390/s24165172

**Published:** 2024-08-10

**Authors:** Fei Gao, Xiaojun Ge, Jinyu Li, Yuze Fan, Yun Li, Rui Zhao

**Affiliations:** 1 College of Automotive Engineering, Jilin University, Changchun 130025, China; gaofei123284123@jlu.edu.cn (F.G.); gexj23@mails.jlu.edu.cn (X.G.); lijy1522@mails.jlu.edu.cn (J.L.); fanyz23@mails.jlu.edu.cn (Y.F.); 2 National Key Laboratory of Automotive Chassis Integration and Bionics, Jilin University, Changchun 130025, China; 3 Graduate School of Information and Science Technology, The University of Tokyo, Tokyo 113-8654, Japan; li-yun@g.ecc.u-tokyo.ac.jp

**Keywords:** intelligent cockpit, human–vehicle interactions, natural elastic interaction, driving experience

## Abstract

Highly integrated information sharing among people, vehicles, roads, and cloud systems, along with the rapid development of autonomous driving technologies, has spurred the evolution of automobiles from simple “transportation tools” to interconnected “intelligent systems”. The intelligent cockpit is a comprehensive application space for various new technologies in intelligent vehicles, encompassing the domains of driving control, riding comfort, and infotainment. It provides drivers and passengers with safety, comfort, and pleasant driving experiences, serving as the gateway for traditional automobile manufacturing to upgrade towards an intelligent automotive industry ecosystem. This is the optimal convergence point for the intelligence, connectivity, electrification, and sharing of automobiles. Currently, the form, functions, and interaction methods of the intelligent cockpit are gradually changing, transitioning from the traditional “human adapts to the vehicle” viewpoint to the “vehicle adapts to human”, and evolving towards a future of natural interactive services where “humans and vehicles mutually adapt”. This article reviews the definitions, intelligence levels, functional domains, and technical frameworks of intelligent automotive cockpits. Additionally, combining the core mechanisms of human–machine interactions in intelligent cockpits, this article proposes an intelligent-cockpit human–machine interaction process and summarizes the current state of key technologies in intelligent-cockpit human–machine interactions. Lastly, this article analyzes the current challenges faced in the field of intelligent cockpits and forecasts future trends in intelligent cockpit technologies.

## 1. Introduction

Highly integrated information sharing and control coordination among people, vehicles, roads, and cloud systems is the current development direction of intelligent transportation systems. Intelligent vehicles with autonomous driving capabilities are the core units of this transportation system [1]. The intelligent cockpit is a comprehensive application space for various new technologies in intelligent vehicles, aiming to reshape the function of human–vehicle interactions and intelligent emotional relationships, providing drivers and passengers with a multi-functional, multi-sensory, interactive experience that includes the driving control function domain, the ride comfort function domain, and the infotainment function domain. It serves as the gateway for upgrading the traditional automobile manufacturing industry to an intelligent automotive industry ecosystem, representing the optimal convergence of vehicle intelligence, connectivity, electrification, and sharing.

According to the degree of automation, the SAE divides the autonomous driving of intelligent vehicles into six discrete levels [2], with the aim of achieving full-range autonomous driving. The gradual proliferation of autonomous driving technology and the continuous advancement of automation levels have led to a shift in the primary and secondary tasks of drivers in intelligent cockpits. Drivers are able to perform non-driving-related tasks to a greater extent, while their information connectivity has given rise to real-time and efficient interconnected mobility scenarios. This evolution propels the transformation of vehicles from mere “transportation tools” to intelligent “third spaces” [1,3]. Complementing the advancements in autonomous driving and information connectivity, traditional cockpits are rapidly evolving into intelligent cockpits.

China’s Society of Automotive Engineers [3] categorizes the process of intelligent cockpits’ transition from “traditional function realization” to “comprehensive intelligent cognition” into five levels. As the level of intelligence of the cockpits increases, their interaction capabilities progressively enhance. Initially, the cockpit passively responds to the needs of the drivers and passengers by providing functional services, which gradually evolves to them actively perceiving the state of the occupants and executing tasks, offering a personalized and pleasant service. Concurrently, the interaction scenarios expand, with the scope of task execution extending from some in-cockpit scenarios to full external scenarios. Additionally, connected services are progressively optimized, expanding from being limited to the internal functions of the in-vehicle system to integrating with the vehicle–road–cloud-integrated control platform, achieving interconnected services and ultimately integrating into smart cities.

**Figure 1:**
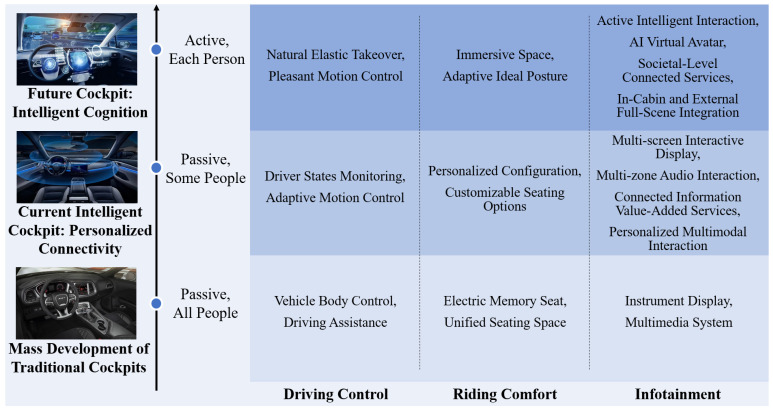
Intelligent cockpit evolution. (Top-down images are from [5,6,7]).

As shown in Figure 1, traditional cockpit design is oriented towards batch development, primarily featuring passive interaction functions, where humans adapt to the vehicles. It lacks consideration for the high-level driving experience demands of users. In terms of driving performance, traditional cockpits focus on power, economy, and driving stability. For riding comfort, they emphasize a standardized spatial layout, seat design, and lighting adjustments. In terms of infotainment, they use mechanical instrument displays, passive button functions, and limited single-machine information sources in their interaction design [4].

Along with continuous technological evolution and the gradual refinement of standards, intelligent cockpits increasingly provide drivers and passengers with safer, more convenient, and more enjoyable personalized and connected experiences. Currently, vehicles are equipped with various sensors to achieve the perception of in-vehicle vision [8]; voice [9], posture [10], and physiological states [11]; vehicle states [12]; and traffic conditions [13], enabling the system to effectively recognize the states inside and outside the vehicle, anticipate demands, and provide intelligent interactions.

In terms of their driving maneuver performance, technologies such as human-like decision making and human-like motion control have been gradually developed to improve driving quality. With respect to ride comfort, technologies for the ideal driving posture, temperature adjustment, and others are progressing gradually, optimizing the ride experience. In infotainment, multimodal interaction technology enhances the ability of the system to serve the user while avoiding the intensification of the driver’s cognitive load caused by single modal interactions [14]. The interconnection of intelligent vehicles with their surrounding physical and virtual environments enables drivers to accomplish more secondary tasks during unmanned driving while facilitating diverse location-based life services. For instance, by utilizing their connectivity, in-vehicle navigation systems can accurately receive real-time updates on traffic congestion and traffic light statuses, enabling vehicles to plan routes more effectively and providing timely and relevant instructions to the driver, such as during stops at traffic lights [15].

The intelligent cockpit is built on essential technologies, structured in layers from the hardware support to the interactive service layer. It incorporates advancements from artificial intelligence, materials science, information communication, sensing technology, the Internet of Things, virtual reality, and augmented reality. These technologies enhance the cockpit’s ability to perceive, understand, and cater to human needs across all scenarios and dimensions. This comprehensive capability fosters acceptance and trust among drivers and passengers, enhancing both functional reliance and emotional attachment. Ultimately, it offers users a sense of freedom, pleasure, and aesthetic satisfaction.

Human–machine interaction is pivotal in the intelligent cockpit’s capability to serve users. The evolution of this interaction has progressed through various stages—from manual controls and graphical user interfaces to the current era of intelligent, multimodal, and multimedia interfaces [16]. The core mechanism of this advanced interaction involves the acquisition of multimodal spatio-temporal data, which includes sensing vehicle dynamics [17], in-cockpit personnel, and external environmental conditions. This comprehensive perception allows for an in-depth understanding of the current actions, behaviors, and emotional states of the vehicle, its occupants, and external traffic participants [18]. By integrating this information with historical data, the system can anticipate the behavioral intentions of the occupants. Consequently, it offers multifaceted, multisensory interactive decision-making services that enhance driving control, comfort, and entertainment.

**Figure 2:**
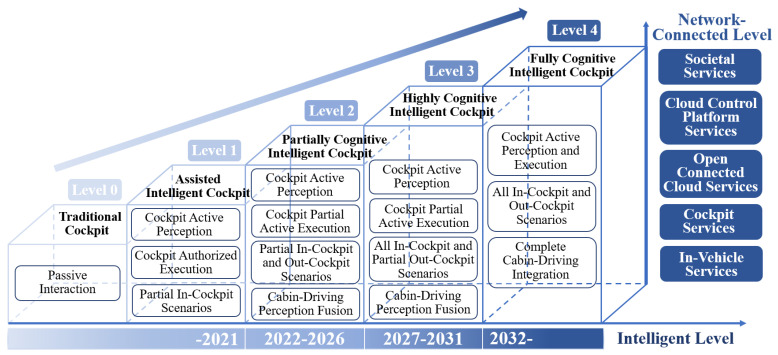
Blueprint for the development of intelligent cockpits.

The future of intelligent cockpits is set to progress towards personalized and adaptable interactions that seamlessly integrate human and machine elements. Figure 2 depicts the developmental blueprint for these advanced cockpits. Affective computing technology is increasingly being integrated into intelligent vehicles, allowing them to clarify uncertainties and ambiguities in raw data, recognize user emotions, and make decisions that cater to emotional dynamics. This enhances the system’s capacity to understand and respond to users effectively [19].

As the link for communication between vehicles and humans, the intelligent cockpit is currently receiving widespread attention from industry, academia, and research communities. In the process of development, the design of intelligent cockpits still faces opportunities and challenges. Key research areas and difficulties for future cockpits include the design paradigm for natural elastic human–machine interaction, quantitative evaluation system for acceptability, quality of interactions, and experience of interactions, and the assurance of safety in coordinating primary and secondary tasks through cabin-driving integration. In this context, natural elastic human–machine interaction refers to a flexible and adaptable interaction within intelligent systems, where interactions between humans and vehicle systems can be dynamically adjusted based on the context, user needs, and environmental conditions, while responding in an intuitive manner that aligns with human habits.

This study focuses on a current full-process development framework for intelligent cockpits, exploring multifaceted challenges and future directions. It builds upon and extends several relevant previous surveys. Li et al. [1] described the fundamental theories and comprehensive evaluation of cockpits but lacked guidance for the development of intelligent cockpit technologies. In another study, Li et al. [13] focused on the development and exploration of cockpit functionalities related to emotion recognition. Murali et al. [20] and Tan et al. [21] concentrated on non-proactive cognitive interactions, specifically highlighting the perception and interaction of driver states in a single-sensor environment, rather than using user-centered feedback and an automated evolutionary cycle of development and guidance within a systematic framework.

However, current intelligent cockpits are expected to be used for multimodal collaboration and are closely integrated with advanced driver assistance systems and vehicle bodies, making them systems that integrate driving control, riding comfort, and infotainment across multiple channels and functionalities. Additionally, vehicle–cloud collaboration empowers natural elastic interactions for intelligent cockpit users, necessitating the exploration of a new, cross-domain, user-centered, and a positive-feedback-driven full-process human–machine interaction development framework to guide cockpit design. No previous reviews have explored this topic. Furthermore, unlike conventional human–machine interaction challenges, intelligent cockpits dedicated to facilitating proactive user interactions and services face unique challenges. For example, the hidden spatio-temporal causality between user behavior sequences and interaction intentions poses challenges to the natural resilient adaptation of human–machine interactions in the cockpit. Based on the current need for new architectural frameworks in intelligent cockpit development and the potentially transformative impact of emerging technologies on cockpit technologies, this study initially reviews basic conceptual classifications of cockpits, proposes a technological support framework, and innovatively presents a novel user-centered, latest full-process human–machine interaction framework, along with insights into the challenges and future directions for cockpit development.

To bridge the aforementioned gaps, this study presents an extensive survey of intelligent cockpits and their interaction technologies. The key contributions are threefold:

- A new full-process human–machine interaction framework is explored. This systematical framework encompasses multimodal perception, cognitive decision-making, active interaction, and evolutionary evaluation. It guides a user-centered, positive feedback-driven, iterative cycle of human–machine interaction to address the complexity and uncertainty of interactions in the original cockpit’s mixed state.
  The framework aims to enhance the efficiency and effectiveness of interactions, ensuring that drivers receive accurate and timely information in complex environments, thereby optimizing the overall driving experience and safety.
- A quantitative evaluation of user experience in intelligent cockpits is described. This study highlights that the quantitative evaluation of user experience primarily focuses on passengers’ subjective experience and objective data analysis based on ergonomics. This aids in forming a user-centered feedback and an automatic evolutionary system.
- Deep insights into the future of intelligent cockpits are provided. Based on the current development status and challenges faced by cockpits, this study innovatively proposes the integration of emerging technologies (such as digital twinning, large language models, and knowledge graphs) as future research directions for intelligent cockpits to better understand user needs.
  For example, a knowledge graph of human–machine interactions can be constructed to mine sparse associative data to determine driver intentions, and this can be combined with large language models to better understand the drivers’ needs and achieve precise predictions of human–machine interaction intentions in intelligent cockpits, as well as to enhance the accuracy of intelligent service recommendations and other downstream tasks.

**Figure 3:**
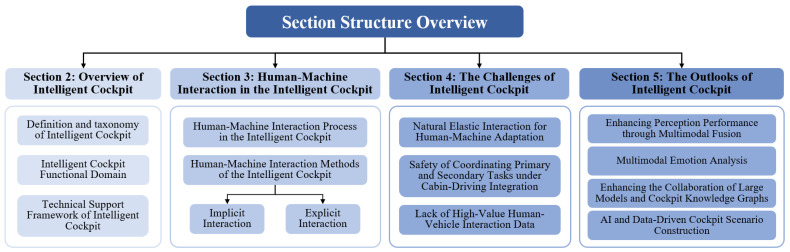
The structure of this study.

The remainder of this article is organized as follows, as shown in Figure 3. Section 2, starting with the definition and intelligence levels of intelligent cockpits, introduces the functional domains and technical support frameworks of intelligent cockpits. Section 3, based on the core mechanisms of human–machine interaction in intelligent cockpits, summarizes the recent interaction technologies from the dimensions of sensing and directing interactions. Section 4 and Section 5, respectively, introduce the challenges faced by intelligent cockpits and future development trends, analyzing the current problems in the field of intelligent cockpits, discussing the solutions to these problems, and exploring the future development trends of intelligent cockpit technology. Section 6 summarizes the main content of this study.

## 2. Overview of Intelligent Cockpits

### 2.1. Definition and Taxonomy of Intelligent Cockpits

An intelligent cockpit, integral to intelligent connected vehicles, combines advanced hardware and software systems to offer robust human–machine interaction capabilities [1]. These capabilities extend across cabin-driving integration, human–machine fusion, scenario expansion, and comprehensive services, providing an enriched experience for occupants. The intelligent cockpit is structured into three technological dimensions: human–machine integration, scenario expansion, and connected services. According to China SAE [3], cockpits are classified into five levels, with each level’s core attributes aligned with these dimensions, as depicted in Table 1. This table outlines the taxonomy of intelligent cockpits. In terms of human–machine interaction, the dimension is articulated through the perceptive and executive subjects involved in cockpit tasks, with higher levels indicating more potent interactive capabilities. For connected services, the evolution from traditional in-cockpit entertainment to broader social-level services marks a significant enhancement of the cockpit’s service capabilities. In the scenario expansion dimension, cockpit tasks are distinguished between in-cockpit and out-cockpit scenarios, with various levels denoting the scope of task execution in expandable scenarios [1].

(1) **Level 0—Traditional Cockpit**
  The task execution in in-cockpit scenarios involves the cockpit passively responding to the needs of the driver and passengers. It offers vehicle system services, including navigation, applications, and phone functionalities, catering to the occupants’ requirements.
(2) **Level 1—Assisted intelligent cockpit**
  The task execution in in-cockpit scenarios can involve active engagement from the cockpit, where it has the capability to perceive the needs of the driver and passengers. This process requires driver authorization and encompasses providing cabin services directly to the occupants. Examples include actively adjusting the air conditioning temperature and airflow, as well as managing related queries and actions to enhance comfort and convenience.
(3) **Level 2—Partially Cognitive Intelligent Cockpit**
  The task execution spans both in-cockpit and selected external scenarios. In certain in-cockpit situations, the cockpit is capable of actively sensing the needs of the driver and passengers. The tasks can be actively executed, in part, with capabilities that link perception to the autonomous driving system for some external scenarios. Furthermore, it facilitates open connected cloud services. Examples include adaptive adjustments to air conditioning and seating based on driving conditions and personal preferences, health monitoring reminders, and push notifications from cloud services.
(4) **Level 3—Highly Cognitive Intelligent Cockpit**
  The task execution can take place in both in-cockpit and selected external scenarios. In all in-cockpit scenarios, the cockpit is capable of actively perceiving the driver and passengers. The tasks can be partially autonomously executed, incorporating perception with the autonomous driving system in most out-cockpit scenarios. Moreover, it supports enhancements to the cloud control platform service. For instance, with Level 2 capabilities, it facilitates cloud control services.
(5) **Level 4—Fully Cognitive Intelligent Cockpit**
  The task execution can occur across all in-cockpit and external scenarios. In every in-cockpit scenario, the cockpit is equipped to actively perceive the driver and passengers. Tasks can be fully autonomously executed, achieving seamless integration with the autonomous driving system in perception, decision-making, planning, and control. Additionally, it supports ongoing upgrades to cloud control and facilitates social-level connected services, including comprehensive cabin-driving integration and proactive cognitive interaction.

### 2.2. Intelligent Cockpit Functional Domain

The functions of the intelligent cockpit are organized into three main dimensions: the driving control functional domain, the riding comfort functional domain, and the infotainment functional domain.

The driving control functional domain is dedicated to enhancing the driving quality of intelligent vehicles. This includes adaptive adjustment technologies for the chassis and suspension that dynamically respond to road conditions and driving behaviors to improve stability. It also features health state monitoring of drivers and passengers, such as fatigue and heart rate monitoring, to ensure safety. These monitoring functions assess the physical condition of drivers and passengers in real time, enabling timely interventions in driving control when potential health risks are detected. Additionally, the dynamic takeover function allows the intelligent cockpit to automatically assume control of the vehicle if insufficient driver response is detected, thereby preventing potential accidents. Furthermore, human-like decision-making and planning processes ensure that the vehicle’s driving path aligns with the occupants’ expected path.

In the paper, the dynamic takeover function is described as a feature of the intelligent vehicle’s driving system, capable of intervening in user operations and executing automated driving control. This is facilitated by multi-dimensional monitoring and judgment of the user, which involves analyzing both past and real-time situational data to predict imminent actions of the user or vehicle. Notably, Nissan’s “brain-to-vehicle” technology [18] uses a headset to collect brainwaves, monitoring the user’s state and predicting driving intentions. This technology can preemptively determine actions such as turning or braking, allowing the intelligent vehicle to execute these actions early and intervene in driving operations. Toyota [22] employs cameras within the cockpit to monitor the driver’s eye and head movements, assessing the driver’s condition. If the driver appears drowsy, the intelligent car takes over the driving tasks to maintain safety. Additionally, HYUNDAI MOBIS’s intelligent cockpit [23] features bio-sensing technology that detects physiological information like the user’s heartbeat, displaying it on-screen. If an anomaly is detected in the user’s physical condition, the system can take control of the vehicle immediately and send distress signals to hospitals or other institutions.

The riding comfort functional domain is centered on enhancing the passenger experience. It includes features like automatic adjustments of the ambient temperature and seat positions, tailored to changes in both internal and external temperatures, as well as to the preferences and emotions of passengers, all aimed at boosting comfort and pleasure. Additionally, this domain offers adjustable ambient lighting and systems for monitoring and regulating air quality inside the vehicle. These features work together to foster a healthier and more enjoyable in-vehicle environment.

The infotainment functional domain delivers a wealth of informational, value-added services and entertainment experiences. For instance, active navigation service recommendations combine traditional maps and traffic data with real-time updates from social media and event information to guide drivers along the best possible routes. Virtual reality immersive experiences can be offered through in-cockpit displays or specialized glasses, enabling passengers to enjoy virtual tours or entertainment during their travels. Additionally, multi-screen linkage displays within the cockpit allow passengers to seamlessly share content across various screens. The interconnection of the intelligent cockpit with both the surrounding physical and virtual environments also facilitates the completion of secondary tasks during autonomous driving and supports a range of location-based lifestyle services.

### 2.3. Technical Support Framework of Intelligent Cockpit

**Figure 4:**
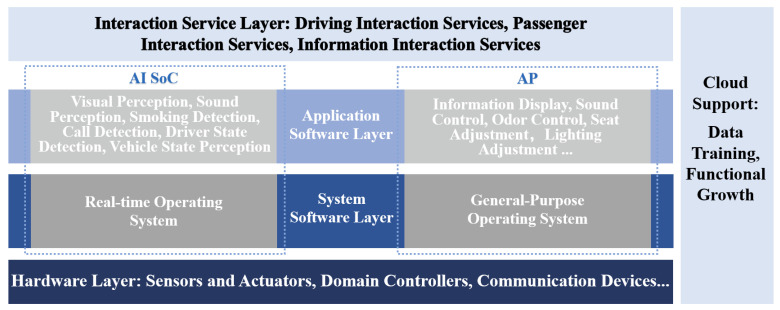
Technology support framework for intelligent cockpits.

The intelligent cockpit is structured in multiple layers from the bottom up, comprising the hardware support layer, the system software layer, the functional software layer, and the interactive service layer. It systematically integrates computing platforms and peripheral devices from both inside and outside the cockpit, along with system software, functional software, and interaction service software. Through continuous learning and iterative improvements, the intelligent cockpit adapts to user needs and emotions across various application scenarios, offering intelligent, convenient, and enjoyable interactive services, as depicted in Figure 4.

Hardware serves as the foundational support and computational backbone for the intelligent cockpit to deliver services. It encompasses sensors and actuator peripherals, domain controllers, cloud platforms, as well as in-vehicle and external interconnection networks. Sensors and actuator peripherals play a crucial role in perceiving and executing various operations and environmental information both inside and outside the cockpit. The sensors commonly employed in current intelligent cockpits include the following [24,25,26,27]:

- Visual sensors. These are installed both inside and outside the cockpit to capture comprehensive visual information. Inside, they record details like the facial expressions and body postures of drivers and passengers, as well as other in-cockpit scenarios. Outside, they monitor environmental conditions such as weather and the behavior of other traffic participants.
- Auditory sensors. These encompass microphones and other devices that are specifically designed to capture auditory information from within the cockpit, such as conversations and ambient noises.
- Haptic sensors. These are integrated into components like the steering wheel, seats, interactive screens, and buttons. They are used to capture information such as pressure exerted by the fingers and bodies of the driver and passengers.
- Olfactory sensors. These are designed to capture olfactory information, such as the various smells within the cockpit.
- Physiological sensors. These capture physiological information about the driver, including electromyography signals, electroencephalography signals, electrocardiography signals, and skin conductance signals.

The main actuators in the intelligent cockpit include the following:

- Visual actuators. These include displays, instrument panels, and head-up displays, all integral to providing visual information directly to the occupants.
- Auditory actuators. These encompass multi-zone speakers and sound processors that enhance the auditory experience within the cockpit by delivering clear and customized audio outputs.
- Haptic actuators. These comprise haptic feedback devices, temperature control systems, and seat angle control mechanisms, all designed to enhance physical comfort and feedback within the cockpit.
- Olfactory actuators. These include fragrance sprayers and air freshening devices, designed to manage and enhance the olfactory environment within the cockpit.

The domain controller serves as the central control unit in the intelligent cockpit, tasked with overseeing and orchestrating the operations of various sensors and actuators. It also handles data processing and task management within the cockpit. In addition, the cloud platform offers expansive computing and storage capabilities, facilitating the deployment of large-scale models and the advancement of cockpit software functionalities. Conversely, the in-vehicle and external interconnection network links different hardware devices within the intelligent cockpit and facilitates communication and data exchange with the external systems [28,29].

As a pivotal element linking hardware and applications, the operating system is crucial for ensuring optimal performance in the intelligent cockpit. It requires high stability, reliability, and security to maintain normal system operations and safeguard user privacy. The intelligent cockpit encompasses a range of functional demands, including entertainment, vehicle control, and navigation systems, necessitating an operating system that is both flexible and scalable to facilitate the operations and interactions of various functional modules.

The cockpit’s diverse functional applications necessitate the coexistence of multiple operating systems, encompassing both safety-critical and non-safety-critical types [30,31]. Safety-critical systems, which manage vehicle dynamics and safety features, typically employ a real-time operating system like QNX OS to ensure swift and predictable response times. Meanwhile, non-safety-critical functions, such as infotainment and connectivity, may utilize more flexible, general-purpose operating systems designed for enhanced user interface and multimedia capabilities, such as Automotive Grade Linux (AGL) and Android OS [30]. Virtual machine technology plays a key role in integrating and securely isolating these operating systems [30], enabling the intelligent cockpit to simultaneously deliver safe driving and rich interactive experiences.

## 3. Human–Machine Interaction in Intelligent Cockpits

### 3.1. The Human–Machine Interaction Process in Intelligent Cockpits

Human–machine interaction is fundamental to an intelligent cockpit, characterized by a functional mechanism that operates in four primary steps: initially, data pertaining to the vehicle and user are collected through sensors located both inside and outside the cockpit [32]. Next, these diverse data are processed and analyzed to predict the user’s needs in the current context. Subsequently, services are delivered to the user through suitable interaction methods [13]. Finally, feedback on these interactive services is gathered from the user to refine the cockpit’s capabilities in perception, prediction, and interaction [1].

**Figure 5:**
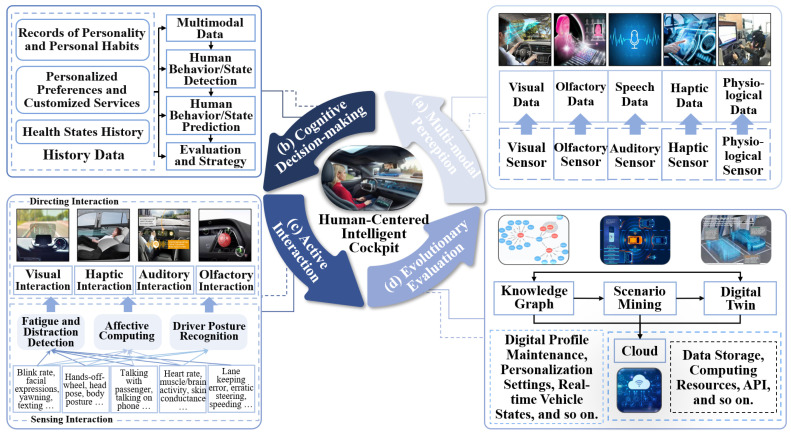
Functional architecture for the process of human–machine interaction. (Images in part (**a**) are from [33,34,35]. Images in part (**c**) are from [36,37,38]. Images in part (**d**) are from [39,40,41].)

From the standpoint of the intelligent cockpit’s functional mechanism, this study introduces a human-centered interaction process architecture. Illustrated in Figure 5, it encompasses four modules: multimodal perception, cognitive decision-making, proactive interaction, and evolutionary evaluation. These modules enable the intelligent cockpit to actively perceive and interpret user intentions, thereby facilitating an informed feedback mechanism that enhances user experience.

Multimodal perception functions are used as the foundational information input system within this intelligent cockpit framework to facilitate human–vehicle interaction [13]. This module’s primary objective is to assimilate and process information from both the internal and external environments of the cockpit via an array of sensors that monitor vehicle dynamics, occupant status, and external conditions. The operational essence of this module lies in its dual ability to gather sensor-derived data and extract valuable multimodal insights. These data are pivotal in furnishing the cognitive decision-making layer with comprehensive datasets that are necessary for advanced analysis and processing. Within the context of an intelligent cockpit, the perception module empowers a thorough understanding of the immediate environment, thereby enhancing the decision-making algorithms, which, in turn, refine user interaction and system responsiveness.

The cognitive decision-making layer represents a critical component of an intelligent cockpit’s human–machine interaction system. Its core functionality revolves around interpreting the multimodal data received from the perception module. This involves analyzing the behavior patterns, current states, and environmental contexts of both drivers and passengers. By leveraging historical data and observed behavioral sequences, this module proficiently predicts the intentions and needs of the users. Subsequently, it tailors the interactive elements and operational modalities of the cockpit to align with user expectations and requirements. This bespoke approach ensures that the system’s responses are both relevant and timely, thereby enhancing the overall user experience within the intelligent vehicle ecosystem.

The multimodal data transmitted from the perception module include user data, dynamic in-cockpit scenario data, and data on the external dynamic environment. The detection of the driver and passengers mainly includes fatigue detection [42,43,44], distraction detection [45,46], workload detection [47,48], emotion detection [14,19,49], and intention recognition [50]. In this context, workload detection and distraction or fatigue detection are related but distinct areas of study. Fatigue detection specifically focuses on the physiological state of the driver, which could lead to decreased alertness or performance. Distraction detection primarily deals with the driver’s focus and attention on elements other than driving tasks. In contrast, workload detection encompasses a comprehensive assessment of how much the driving task or associated tasks demand from the driver cognitively and physically, which could overlap with the effects observed in fatigue and distraction scenarios but is a broader measure. Wei et al. [48] introduced a method for classifying driver mental workload by quantifying multiple factors, including physiological signals, traffic conditions, and environmental variables. Meanwhile, Wang et al. [47] developed a deep learning approach to assess cognitive load in drivers of SAE L3 vehicles. This method utilized a range of physiological indicators, such as electrocardiograms, electrodermal activity, and respiratory patterns, and it employ a transformer–encoder-based network to account for the temporal correlations in the data. Predicting user intentions based on their state and dynamic scenarios requires historical data, which mainly include user health history, past behavior, and personalized preferences. Through state assessment and intention prediction, functional features are triggered, generating decision strategies accordingly. The cognitive decision-making module is a critical link in an intelligent cockpit system. The accuracy and efficiency of the algorithms in this module directly determine the performance and user experience of intelligent cockpit systems.

Active interaction is the execution phase of the human–machine interaction system in an intelligent cockpit. After the cognitive decision-making module completes its assessment based on the current context and generates a strategy, it outputs this strategy to the interaction module. The task of the interaction module is to execute the interactions between the cockpit and the user, as well as the interactions between the cockpit and the external environment, providing the user with multidimensional services related to driving, riding, and information.

The evolution evaluation module collects information reflecting user satisfaction and experience from the interaction phase, quantifies the user’s evaluation of proactive interaction services, and twins interaction scenarios to further evolve models and methods to enhance the intelligent system’s prediction and decision-making capabilities. Quantitative evaluation and enhancement of user experience are key factors in guiding the improvement of the performance of intelligent cockpits. User experience can be quantitatively evaluated using various metrics and methods, such as subjective and objective evaluations [51,52,53] of intelligence, safety, efficiency, and pleasure.

The quantitative evaluation of user experience primarily focuses on the subjective experience of passenger comfort and objective data analysis based on ergonomics, thereby forming a passenger-centric loop of feedback and automatic evolution. The subjective evaluation methods for ride comfort intuitively represent the riding experience of passengers. For example, Huang et al. [54] utilized a ten-point scale to assess the comfort performance of vehicles with different seat lumbar supports at various speeds to explore optimal seat designs. Similarly, Cardoso et al. [55] employed perceived discomfort ratings and automotive seat discomfort questionnaires to comprehensively assess the discomfort levels of subjects to aid in the optimization of seat designs in commercial vehicles.

However, subjective evaluations are constrained by the professional experience and assessment capabilities of evaluators, resulting in randomness in the assessment outcomes. In contrast, objective evaluation methods focus on objective data that represent information on the state of the human body. These methods use sensors or measurement instruments to detect objective data such as the motion behaviors and physiological states of passengers. By combining data processing and algorithmic approaches such as inductive statistics, these methods quantitatively analyze the objective burden on passenger comfort. Techniques based on pressure distribution [56] evaluate and optimize seat comfort by analyzing the pressure distribution in the contact area between the passenger and the seat. Wolf et al. [57] calculated the optimal driving postures for three different driving load scenarios based on the angles of six joints—the ankle, knee, hip, humerus, elbow, and pelvis—and qualitatively verified the significant effects and preference trends of individual factors such as age, gender, and body size on joint angles for personalized comfort [58] by measuring joint angles in the most comfortable driving postures for different subjects. Physiological electrical signals from passengers can directly represent their physiological burden, thus revealing the mechanisms of discomfort and serving as a basis for assessing ride comfort. Gao et al. [59] studied the effects of seat cushion and backrest angles on muscle activation levels and muscle force to determine the optimal recommended angles for cushions and backrests. Lecocq et al. [60] collected electromyographic signals from the main working muscles of drivers during long driving sessions to explore the impact of seat firmness on muscle activation across different body parts and on ride comfort. Objective evaluation methods offer high accuracy, and their experimental designs are highly reproducible. However, the drawbacks include the diversity of objective indicators, reliance on statistical induction and parametric comparisons, and the complexity of the methodologies used.

The integrated quantitative assessment method, which combines both subjective and objective measures, mines the mapping relationships between objective indicators, such as pressure distribution and physiological electrical signals, and subjective comfort levels. This method involves the construction of a quantitative digital evaluation model that can generate scores with high consistency with subjective sensations based on objective parameters with the aim of assisting in the optimization of key human–machine interaction components in intelligent cockpits. Tang et al. [61] measured the impact of differences in seat cushion shapes on the pressure distribution across the hips and thighs and integrated these findings with subjective evaluations to optimize seat comfort designs. Li et al. [62] investigated the pressure distribution on the buttocks, thighs, and back under short and long driving conditions, and established a subjective–objective mapping relationship using the analytic hierarchy process (AHP) with entropy weighting. Wang Dawei et al. [63] developed a comfort prediction model based on multimodal physiological information using an improved BP neural network. This integrated quantitative assessment method blends the strengths of both subjective and objective evaluations, offering high personalization, accuracy, and objectivity.

With the development of large models and data analysis technologies, the design of an intelligent cockpit integrates the detailed management of personalized user needs and experiences, continuously improving the experience in driving control, riding comfort, and infotainment functions. This is intended to provide drivers and passengers with intelligent, safe, efficient, and pleasant experiences. The system continuously accumulates historical data, enhancing its ability to judge and predict, forming a user-centered loop of feedback and automatic evolution.

### 3.2. Human–Machine Interaction Methods of the Intelligent Cockpits

In recent years, human–vehicle interaction technology has advanced significantly, enhancing the cockpit experience. Interaction methods have evolved from traditional in-vehicle displays and basic voice dialogues to more sophisticated, intelligent interactions. These include integration of graphical user interface (GUI) displays, auditory [64], tactile [65], air gestures [66], wearable sensors [67], and augmented reality (AR) technologies [68]. Such systems are capable of predicting and inferring the user’s behavior, cognitive state, and emotions [69], thereby enabling proactive interaction and evolutionary feedback.

**Figure 6:**
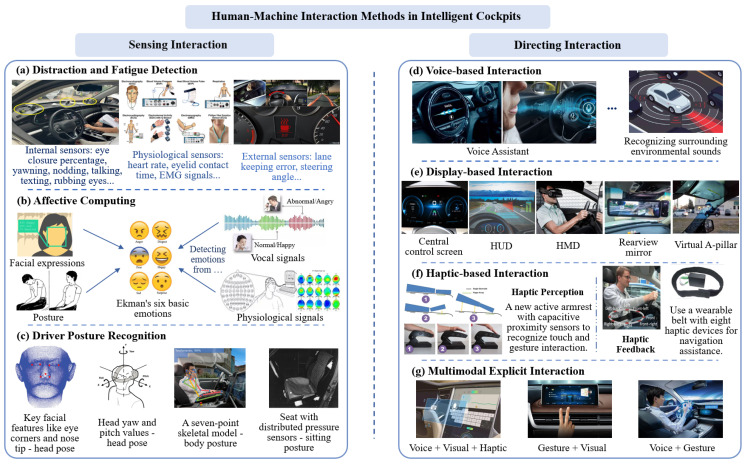
Human–Machine Interaction Methods of the Intelligent Cockpit. (Images in part (**a**) are from [70,71]. Images in part (**b**) are from [72,73,74]. Images in part (**c**) are from [75,76,77,78]. Images in part (**d**) are from [79,80,81]. Images in part (**e**) are from [82,83,84,85,86]. Images in part (**f**) are from [87,88]. Images in part (**g**) are from [89,90,91]).

This section reviews the latest interaction technologies in intelligent cockpits, presenting a detailed analysis from the perspectives of sensing and directing interactions [20]. These developments are illustrated in Figure 6, highlighting the diverse technologies that contribute to a more responsive and immersive cockpit environment.

#### 3.2.1. Sensing Interaction

Sensing interaction is centered on the indirect interactions with the user by capturing and analyzing non-specific behaviors like body movements, physiological signals, facial expressions, and environmental factors. This capability enables the system to intelligently discern the user’s current state and needs, allowing for spontaneous responses [20]. This section delves into the pivotal technologies underpinning sensing interaction, covering areas such as driver fatigue and distraction recognition, affective computing, and driver posture detection. These technologies are crucial for enhancing user interaction by making the system more attuned to the subtle cues of the user’s physical and emotional state, as detailed in Table 2.

(1) **Driver Fatigue and Distraction Detection**

Inattention in driving refers to the diminished focus on essential safe driving activities, occurring even in the absence of overt distractions [92]. Distraction and fatigue are commonly identified as leading contributors to driver inattention [93,94]. The research indicates that driver distraction ranks among the primary causes of road traffic accidents [95]. Distractions can arise actively, when drivers use their hands, eyes, or thoughts for secondary tasks, thereby diverting attention away from the main task of driving [96,97,98]. Additionally, distractions can occur passively, such as being inadvertently drawn away by auditory stimuli.

Driver distraction detection methods often rely on sensors such as inertial measurement units (IMUs) [99], GPS [100,101], and external cameras [102,103] to monitor the vehicle’s state in real-time. These sensors track metrics like vehicle speed, yaw angle, and lane keeping to assess the stability of driving patterns, which can help indirectly identify whether the driver is distracted. Typically, the vehicle speed data are integrated with other sensor outputs to evaluate the driver’s attention state. For example, Greenberg et al. [104,105] used lane position and lane-keeping errors as markers to detect driver distraction. Zhang et al. [106] applied binary logistic regression and Fisher discriminant analysis to differentiate between normal and distracted driving based on lane-keeping errors. Additionally, Nakayama et al. [107] employed steering wheel sensors to predict steering angles using a second-order Taylor series and measured steering errors to infer distraction.

Compared to external sensors, in-vehicle visual sensors such as stereo cameras and infrared cameras are extensively used to monitor driver and passenger behaviors. These visual sensors are frequently paired with deep neural network architectures to leverage neural networks for identifying driver behaviors from single images. Utilizing convolutional neural networks, Eraqi et al. [108] applied genetic algorithms to optimize classifier weighting, successfully detecting and categorizing distraction activities like making calls, texting, looking away from the road, and rubbing eyes. Tran et al. [109] performed a comparative analysis of various neural network architectures, including VGG, AlexNet, GoogleNet, and ResNet, for driver distraction detection. They proposed an end-to-end network based on the pre-trained VGG-19 architecture, which effectively adjusts to variations in brightness and shadows, thereby accurately detecting driver distraction behaviors.

**Table 2:** Sensing interaction methods.

Category	Data/Data Source		Works	Methodology
Distraction and Fatigue Detection	External Sensors		[104,105]	Detect driver distraction through lane position and lane-keeping errors.
			[106]	Differentiate between normal and distracted driving based on binary logistic regression and Fisher discriminant analysis.
			[107]	Use steering wheel sensors to calculate steering error and indirectly determine if the driver is distracted.
	In-vehicle Visual Sensors		[108]	Classify distraction behaviors using a weighted ensemble of CNN classifiers with genetic algorithms.
			[109]	Utilize convolutional neural networks (CNN) to effectively adapt to changes in brightness and shadows.
			[110]	Utilize support vector machines (SVM) to detect eye closure, thereby detecting distractions and other behaviors.
	Physiological Sensors		[111]	Utilize SVM to classify EEG signals during driving into four categories.
			[112]	Use in-ear EEG instead of traditional head electrodes.
			[113,114,115]	Use ECG to detect driver behavior linked to heart rate, like emotions.
			[116]	Utilize EOG sensors to record eye movements, such as eyelid contact time, for detecting fatigue and distraction.
			[117,118,119]	Utilize EMG to detect changes in muscle signals to determine fatigue.
Affective Computing	RGB or IR Cameras		[120,121,122]	After collecting facial data using RGB or IR cameras, accurately classify the data using k-nearest neighbors (k-NN), SVM, and deep neural networks.
			[123,124,125]	
			[49,126]	
	Physiological Sensors		[127,128]	Generate physiological signals with sensors, reduce dimensions with PCA and LDA, then classify with SVM and naive Bayes.
			[129,130]	
	Voice Signals		[131]	Use features of voice signals (such as pitch, energy, and intensity) for emotion recognition.
			[14]	Integrate global acoustic and local spectral features of voice signals to capture both overall and instantaneous changes in emotions.
	Driver Behavior		[132]	Distinguish driver emotions by detecting steering wheel grip strength.
			[133]	Detect driver frustration through posture using SVM.
	Multimodal Data		[134]	Integrate gestures and voice for emotion analysis.
			[135]	Utilize convolutional techniques to input drivers’ facial expressions and cognitive process features for emotion detection.
			[136]	Classify emotions for each modality using a Bayesian classifier, then integrate at the feature and decision levels.
			[137]	Using facial video and driving behavior as inputs and employing a multi-task training approach.
Driver Posture Recognition	Visual Sensors	RGB Camera	[138]	Use template matching methods for head pose estimation and tracking.
			[139]	Use a quantized pose classifier to detect discrete head yaw and pitch.
			[75]	Estimate head pose by tracking key facial features such as eye corners and the tip of the nose.
		Multi- cameras	[140]	Adopt a multi-camera strategy to overcome the limitations of a single-perspective monocular camera.
		IR Camera	[141]	Estimate head pose using infrared images based on deep learning methods.
		Structured Light	[142]	Based on a graph-based algorithm, use depth frames to fit a 7-point skeleton model to the driver’s body to infer posture.
		Time of Flight	[143]	Based on the iterative closest point algorithm, estimate the position and orientation of the driver’s limbs using visual information.
	Haptic Sensors		[78]	Measure the driver’s posture using optical motion capture and a seat equipped with distributed pressure sensors.
			[144]	Deploy pressure maps integrated with IMU measurements to robustly identify body postures.
			[145]	Accurately estimate the 3D position of the occupant’s head using a capacitive proximity sensor array and non-parametric neural networks.
			[146]	Estimate head pose based on ultrasonic technology.

Analyzing the facial features of drivers can also effectively determine whether they are distracted or fatigued. Craye et al. [110] employed machine learning techniques such as support vector machines (SVM) to analyze eye closure. Additional indicators used to identify fatigue and distraction include the percentage of eyelid closure over time (PERCLOS) [110], the duration of gaze deviation from the road [147], and behaviors such as yawning and nodding [110,148]. Moreover, internal cameras can ascertain whether both hands are off the steering wheel, and infrared cameras help mitigate the effects of lighting changes.

In addition to visual sensing technology, physiological sensors such as electromyography (EMG), electroencephalography (EEG), electrocardiography (ECG), and electrooculography (EOG) are also utilized to detect driver distraction behaviors. Yeo et al. [111] employed SVM to classify EEG signals into four categories during driving. However, the placement of EEG electrodes can limit its widespread use. Hwang et al. [112] discovered that in-ear EEG achieves accuracy comparable to single-channel EEG in classifying driver alertness and fatigue. Additionally, as driver emotions and physical exertion influence heart rate, ECG can provide valuable data for monitoring these states [113,114,115]. EOG is used to record eye movements, such as eyelid contact time, which assists in detecting fatigue [116]. Furthermore, the amplitude of EMG signals tends to decrease during fatigue [117,118,119]. Although physiological sensors are inherently invasive, advancements in brain–machine interface technology, like Neuralink’s approach to directly implant wireless devices into the skull, could offer more detailed insights into brain activity relevant to driving.

(2) **Affective Computing**

Affective computing is designed to enable computers to recognize and emulate human emotional characteristics, fostering natural and intimate interactions with humans. This technology concentrates on interpreting and responding to the driver’s emotional state. Accurately understanding these emotions is vital for optimizing human–vehicle interaction, as it can significantly enhance the responsiveness and adaptability of the system to the driver’s needs and conditions.

Emotion recognition is a fundamental component of affective computing. It plays a critical role not only in detecting driver distraction and fatigue [149] but also in providing detailed driving information [150,151] for intelligent vehicles. Ekman et al. [152] highlighted that basic emotions—such as anger, disgust, happiness, sadness, surprise, and fear—expressed through facial expressions, exhibit cross-cultural universality [153]. These emotions can be accurately classified using methods like k-nearest neighbors (k-NN) [120,121,122], support vector machines (SVM) [123,124,125], and deep neural networks [49,126] after facial data are captured with RGB or infrared cameras. Furthermore, physiological signals serve as essential data for emotion recognition. Despite their high-dimensional and time-varying nature, these signals can be effectively classified using SVM or naive Bayes after dimensionality reduction techniques such as principal component analysis (PCA) [127,128] and linear discriminant analysis (LDA) [129,130] are applied.

The research indicates that voice signal characteristics like pitch, energy, and intensity are effective in recognizing emotions [131]. Li et al. [14] proposed a multi-feature fusion network that captures both overall and instantaneous emotional shifts by integrating global acoustic properties and local spectral features of voice signals. These instantaneous changes are especially critical for identifying specific emotional states, such as sudden anger or excitement. Additionally, driver behavior offers insights into emotional states. Oehl et al. [132] observed significant differences in steering wheel grip force when drivers were happy compared to when they were angry. Similarly, Taib et al. [133] employed SVM and other machine learning techniques to detect emotions such as frustration based on the driver’s posture.

Compared to relying on single modalities like facial expressions or voice, multimodal emotion analysis leverages synergistic complementarities across different modalities to enhance emotional understanding. Zadeh et al. [134] designed a network that integrates gestures and voice for more comprehensive emotion analysis. Li et al. [135] used convolutional techniques, incorporating drivers’ facial expressions and cognitive process features such as age, gender, and driving experience to develop a driver emotion detection model. Caridakis et al. [136] combined inputs from facial expressions, body movements, gestures, and voice, utilizing a Bayesian classifier to categorize emotions within each modality before integrating features and decisions to accurately recognize eight distinct emotions. Hu et al. [137] improved the accuracy of driver emotion recognition by using facial videos and driving behaviors—including brake pedal force and vehicle Y-axis and Z-axis positions—as inputs, while employing a multi-task training approach.

(3) **In-vehicle Posture Recognition**

In-vehicle posture recognition is a crucial aspect of sensing interaction, utilizing sensors and detection technologies to capture and analyze the body movements and postures of passengers. This system primarily aims to understand the body language and behaviors of the driver and passengers to enhance service delivery and responses.

Head pose estimation and tracking are essential for monitoring the driver, detecting distractions, identifying gaze direction, and calibrating head-mounted devices. Guo et al. [138] extracted features from RGB images and applied a template matching method to estimate and track head poses. Wu and Trivedi [139] utilized a quantized pose classifier to detect discrete head yaw and pitch angles. Martin et al. [75] estimated head poses by tracking key facial features such as the corners of the eyes and the tip of the nose, although this method faces challenges like occlusions and the limitations of a single camera perspective. To address the drawbacks of monocular estimation, Tawari et al. [140] adopted a multi-camera approach within the driver’s field of view. Additionally, Firintepe et al. [141] introduced a method called head direction network (HDN) and ResNetHG to process infrared images for head pose estimation. Structured light depth cameras have also been employed for more accurate driver pose estimation. Kondyli et al. [142] proposed a graph-based algorithm that fits a 7-point skeleton model to the driver using a series of depth frames, while Demirdjian et al. [143] utilized a time-of-flight depth sensor to estimate the position and orientation of the driver’s limbs.

Haptic sensors are increasingly utilized for estimating body and head postures within vehicles. Andreoni et al. [78] experimented with measuring the driver’s sitting posture using a seat equipped with distributed pressure sensors. Similarly, Vergnano et al. [144] robustly identified body postures by integrating pressure maps with measurements from inertial measurement units (IMUs). Ziraknejad et al. [145] employed sensor-equipped headrests with capacitive proximity sensors to estimate head posture and control headrest positioning. Pullano et al. [146] utilized ultrasonic sensors embedded in the headrest for head posture estimation. However, for a more robust estimation of body and head postures, it is beneficial to combine haptic sensors with visual sensors in a multimodal approach, enhancing accuracy and reliability in diverse conditions.

#### 3.2.2. Directing Interaction

Compared to sensing interaction, directing interaction emphasizes users consciously and explicitly expressing their intentions and needs. The hallmark features of this mode of interaction are certainty and directness, which often make it more efficient in various situations. For instance, when a driver needs to quickly alter the navigation destination, direct voice commands can be faster and more accurate than relying on predictive, implicit responses. Directing interaction typically encompasses voice-based, display-based, and haptic-based interactions, which are outlined in detail in Table 3.

(1) **Voice-based Interaction**

Voice-based interaction involves users directly communicating with systems or devices through voice commands or conversations. Compared to traditional methods like touch, buttons, or other physical interactions, it offers a more intuitive and natural interface [196]. The voice user interface (VUI) has become a primary mode of modern human–vehicle interaction. Automakers are continually developing and integrating intelligent voice assistant technologies, such as BMW’s intelligent personal assistant [9] and Mercedes-Benz’s MBUX voice assistant [154]. Digital voice assistants (VA) can accurately recognize and execute driver commands, controlling in-vehicle functions like navigation [155], entertainment [156,157], and communication [158]. They also facilitate vehicle-to-vehicle communication [159,160] by collaborating with other voice assistants. Lenstrand et al. [197] suggest that AI-based voice assistants enhance the user experience by making it more engaging and effective [198].

During long night drives, conversing with other passengers can help drivers stay alert [161]. However, engaging in phone calls increases cognitive load and reduces alertness. Rosekind et al. [161] observed that active participation in a conversation is more effective at maintaining driver alertness than passive listening. Therefore, the design of future voice assistants (VAs) should prioritize natural dialogues with the driver over mechanical, command-based interactions. Using the Wizard-of-Oz method, Large et al. [162] found that brief, intermittent conversations with a VA can significantly enhance alertness. Furthermore, Wong et al. [163] discovered that VAs with a confident tone are more effective in capturing the driver’s attention. Additionally, Wang et al. [164] suggest that female voices are often perceived as more trustworthy, acceptable, and pleasant compared to male voices.

Under Level 3 autonomous driving conditions, timely responses to takeover requests are critical for human–vehicle interaction, ensuring that control can be quickly and safely transferred back to the driver when necessary to maintain road safety. Considering the driver’s situational awareness and distraction levels, voice assistants (VAs) can play a significant role in this process. Mahajan et al. [165] conducted experiments using a driving simulator to compare takeover response times with and without the aid of VAs. The results indicated that VAs can effectively enhance the driver’s efficiency in regaining control of the vehicle.

During driving, the driver may not always detect the sounds of nearby emergency vehicles, such as ambulances, due to distractions or the vehicle’s soundproofing. To address this, sound technology can be implemented to detect these emergency sounds and trigger alerts to enhance driver alertness. Meucci et al. [166] developed a pitch detection algorithm based on signal processing, specifically designed for the real-time identification of emergency vehicle sirens. Tran et al. [167] proposed a model using convolutional neural networks that can effectively distinguish between regular noise, sirens, and other vehicle sounds, thus accurately identifying emergency vehicle sirens.

However, the inherent characteristics of voice user interfaces (VUIs), such as serialization and temporality, introduce delays in VUI responses and feedback. This can lead to user uncertainty about whether the VUI has received their command or is processing the request [199,200]. Moreover, interaction with VUIs often relies on short-term memory [201,202], which can potentially compromise task efficiency and safety, especially when the driver is handling multiple tasks simultaneously.

(2) **Display-based Interaction**

Display-based interaction primarily involves engaging with users through GUIs, displays, or other visual devices. This method leverages visual cues such as icons, text, colors, and other graphical elements to provide users with intuitive and efficient prompts for information and operation. This approach helps ensure that interactions are not only user-friendly but also enhance the overall effectiveness and speed of communication between the user and the system.

Modern vehicles are commonly equipped with a central display unit that incorporates various input methods, including traditional buttons and knobs, touchscreens, voice commands, or gestures [203]. The instrument panel serves as the foundational information display in the cockpit, relaying crucial data about driving conditions and vehicle mechanics to the driver [69], such as vehicle speed and engine RPM. With the rapid advancement of automotive technology, the central control screen has emerged as a vital feature in connected vehicles [204]. As the centralized hub for in-vehicle information, it provides drivers and passengers with access to vehicle statuses, navigation, and entertainment options. Additionally, the central console displays various driving assistance details, including lane departure warnings, collision warnings, and blind-spot monitoring. The central control screen also integrates numerous vehicle control functions, such as adjusting the air conditioning temperature, seat heating, and the operation of windows and sunroofs.

While traditional input methods often require drivers to divert their gaze, technologies like head-up displays (HUD) and head-mounted displays (HMD) help mitigate this by integrating virtual elements with real-world scenes, reducing the need for drivers to look away. Furthermore, the advancement of virtual reality (VR) and augmented reality (AR) technologies has significantly enhanced human–machine interaction. These technologies create immersive, three-dimensional environments that offer novel visual and sensory experiences. They allow users to operate and communicate within virtual or augmented realities more naturally and extend the spatial dimensions of interaction. Users can interact directly with information and virtual objects in a three-dimensional space, moving beyond traditional screen limitations to provide unprecedented freedom in interaction. Integrating AR and VR into HUD or HMD can greatly amplify the effectiveness of interactive experiences.

Park et al. [168,169] proposed an in-vehicle augmented reality head-up display (AR-HUD) system that leverages stereo cameras to capture forward images and utilizes SVM to detect and recognize vehicles and pedestrians on the road in real-time. This system intelligently matches enhanced driving safety information with real-world data on vehicles and pedestrians, based on the number of lanes, road type, vehicle speed, and road attributes, providing timely information through a transparent screen mounted in front of the driver. Additionally, using mobile virtual reality technology, Hock et al. [205] developed CarVR technology, which enables passengers to enjoy an immersive experience within a moving vehicle through a head-mounted display, while also minimizing simulator-induced motion sickness.

To ensure a stable display of virtual elements in HUDs and HMDs, precise head pose tracking and accurate registration are essential. Registration is the process of accurately anchoring virtual elements within the real scene. Gabbard et al. [170] highlighted that achieving accurate registration requires recognizing the posture of the vehicle, the driver’s head and eyes, and external targets. Additionally, to address challenges like vehicle vibrations, driver head movements, and occlusions in the real scene, registration must maintain real-time performance. An et al. [171] proposed a real-time registration algorithm that achieves an average registration time of 0.0781 s, effectively minimizing visual lag. Tasaki et al. [172] suggested a method to temporarily hide virtual elements in the HUD during vehicle vibrations to mitigate the impact of these vibrations on registration accuracy.

Jiang et al. [173] conducted a comprehensive review of AR-based system registration methods. Given the variations in ambient light between daytime and nighttime driving, AR HUDs also require light compensation techniques. Gabbard et al. [174] developed an active strategy that samples background and ambient light to adaptively adjust the brightness and color of the AR HUD content, ensuring that virtual information remains clearly visible to the driver under varying lighting conditions. Broy et al. [175] investigated the use of autostereoscopic displays in vehicle dashboards, finding that quasi-3D displays enhanced performance in auxiliary tasks. However, while complex display interfaces can provide a more immersive experience, they may also distract from the primary driving task. Therefore, a balance must be struck when integrating advanced autostereoscopic display technologies into vehicle systems.

In addition, rearview mirrors, traditionally essential for driving, have been significantly enhanced through technological upgrades, transforming into smart rearview mirrors. These modern mirrors are equipped to display real-time video captured by rear cameras, providing a broader, blind-spot-free view. Malcolm et al. [176] developed a user-centric, camera-based interior rearview mirror that combines the functionality of a traditional mirror with a built-in display. This allows the driver to toggle between using it as a standard glass mirror or as a digital display to see the environment behind the vehicle whenever necessary, thus enhancing both safety and versatility.

In the traditional three-mirror system, vehicles use two side mirrors to display the environment on the left and right sides, while the central rearview mirror is adjusted to allow the driver to see the rear through the vehicle’s rear windshield. Pan et al. [177] noted that this system requires the driver to shift attention among the mirrors to obtain a complete view of the rear environment, which can pose safety challenges. To address these issues, they proposed a rear-stitched view panorama (RSVP) system, which utilizes four rear cameras. This system stitches together images from each camera to create a single panoramic view, displaying the entire rear environment and effectively eliminating traditional blind spots.

The A-pillar is a crucial component of a vehicle’s body structure, positioned at the front, where it connects the roof to the body’s base. While it serves to protect the driver and passengers, the A-pillar also obstructs the driver’s forward view, creating blind spots that can increase the risk of traffic accidents. One approach to enhance visibility through the A-pillar involves designing cutouts with simple, regular hollow shapes [178,179,180]. Additionally, some innovative designs project real-time images onto the A-pillar to show the driver the external environment that is otherwise blocked [86]. Building on this concept, in 2018, the German Continental Group developed the virtual A-pillar, which effectively renders the A-pillar almost transparent, aiming to eliminate forward blind spots and enhance driving safety.

(3) **Haptic-based Interactions**

Haptic-based interaction methods engage the human tactile system to interact with devices or applications, moving beyond traditional touch or press operations. Modern approaches include integrating haptic sensors into armrests and seats, allowing interaction without the need for visual or auditory cues. For instance, the tightening of a seatbelt can serve as an alert to the driver.

Inspired by Hjorth parameters [206], Kaboli et al. [181,182,183] developed a robust haptic descriptor that captures the statistical characteristics of haptic signals in the time domain, making it suitable for diverse sensor environments. Utilizing these haptic descriptors, Kaboli et al. [181,184] employed SVM classifiers to facilitate the combinatory classification of gestures. This approach enables the dynamic selection of specific units from distributed haptic sensors, enhancing the efficiency and accuracy of data processing. Additionally, Kaboli et al. [185,186] demonstrated that haptic sensors can be used to learn, recognize, and distinguish between contacted objects. In another innovative application, Braun et al. [87] employed a novel active armrest equipped with capacitive proximity sensors to estimate arm posture and detect single or multipoint touches, utilizing SVM classifiers to recognize gestures effectively.

Intelligent vehicles utilize sensor perception to interact with users through haptic feedback. Vibration haptic feedback is particularly effective in warning drivers of potential dangers, such as collisions during parking [187], lane departures [188], or speeding [189]. This type of feedback helps heighten the driver’s awareness of their surroundings and enhances safety. Asif et al. [88] employed a wearable belt equipped with eight haptic feedback devices to provide navigation assistance, significantly reducing the cognitive load that often accompanies visual-based navigation systems. Similarly, Hwang et al. [190] enhanced navigational aids by embedding a 5×5 haptic feedback matrix into the seat, allowing for discreet and intuitive directional guidance without requiring visual or auditory input from the driver. These implementations demonstrate how haptic technology can be seamlessly integrated into vehicle systems to improve interaction and safety.

Gaffary and Lécuyer et al. [207] have highlighted that haptic interaction may not be appropriate for all scenarios. Supporting this, research by Nukarinen et al. [208] demonstrated that relying exclusively on haptic feedback for navigation tasks can lead to errors. This is because, while haptic interaction can effectively provide alerts and control commands, it lacks the capability to offer detailed navigation information and instructions. Additionally, the design of the haptic feedback must be carefully considered to prevent startling the driver, which could lead to dangerous situations. To enhance interaction effectiveness, the research is now exploring the integration of haptic feedback with other modalities, such as visual and auditory feedback [209,210,211,212], to create a more comprehensive and effective multi-sensory user experience.

(4) **Multimodal Directing Interaction**

Single-modal interactions, while useful, have inherent limitations. Incorporating multimodal interactions can lead to more effective outcomes. Multimodal interactions combine various types of feedback—visual, auditory, and haptic—to offer a richer and more comprehensive user experience. This approach capitalizes on the strengths of each modality to ensure that critical information is communicated both effectively and efficiently. By doing so, it enhances user engagement and improves the accuracy and speed of the information conveyed, catering to the diverse sensory preferences of users.

Pieraccini et al. [191] explored combining voice with visual or haptic feedback to address the latency issues commonly associated with single-modal voice interactions. Pfleging et al. [192] designed an interaction system that integrates gestures and voice, which helps reduce visual distractions while satisfying the specific needs of driving. Braun et al. [193] investigated multimodal outputs that merge voice and visual feedback. Their findings indicate that enhancing voice user interfaces (VUIs) with visual text and icons can improve the effectiveness of interactions and bolster the user’s short-term memory retention. Furthermore, Braun et al. [193] also explored combining voice commands with tactile inputs such as haptic buttons and knobs on the steering wheel for navigation and for correcting voice-transcribed text. Similarly, Jung et al. [194] developed an interaction framework that utilizes both voice and haptic feedback. Additionally, Lee et al. [195] employed gestures in conjunction with visual aids like head-up displays to facilitate menu control, demonstrating the versatility and effectiveness of multimodal interactions in enhancing user experience and functionality.

## 4. The Challenges of Intelligent Cockpits

**Figure 7:**
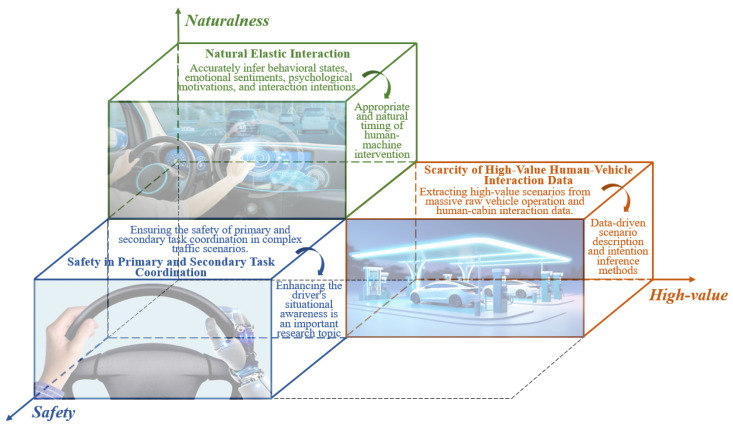
Challenges faced by intelligent cockpits. (images are from [213,214]).

As autonomous driving technology becomes increasingly widespread and information connectivity continues to improve, intelligent cockpits will continue to rapidly evolve. In human–machine interaction, the driver’s primary role is evolving. There was once a focus on driving operations and monitoring surroundings, but now it has shifted towards supporting dynamic driving tasks beyond the system’s scope. Looking ahead, this focus will move towards user-demand-oriented intervention control in multiple adaptive scenarios. In this context, a “dynamic driving task” refers to the vehicle system providing real-time assistance or taking over driving tasks based on changing conditions such as traffic patterns or the driver’s state. Compared with traditional driving operations, the vehicle handles more sophisticated situational responses to enhance safety and driving efficiency, allowing the driver to focus more on supervising the vehicle’s operations rather than directly controlling it. Meanwhile, “user-demand-oriented intervention control” pertains to the vehicle system proactively adapting to the specific preferences and needs of the user in various driving scenarios. This may include adjusting vehicle behavior based on the user’s historical preferences and real-time input to enhance comfort, safety, or efficiency. The role of the secondary task is evolving from the traditional scenario of full human adaptation to the vehicle with “passive service requests” to the current scenario where the vehicle adapts to the human with “proactive service provision,” while moving toward a future of personalized mutual adaptation between human and vehicle in a natural and elastic interactive relationship. This section outlines several challenges faced during the development of intelligent cockpits, as shown in Figure 7.

### 4.1. Natural Elastic Interactions for Human–Machine Adaptation

The continuous elevation of user needs has led to a shift in the positioning of human–machine interaction. Drivers and passengers now demand high safety, high comfort, high social capability, high recognition, and high personalization of vehicles. Accordingly, vehicles must possess data analysis, behavior prediction, and service connection capabilities, as well as the ability to perceive and understand user emotions and intentions, to provide natural and elastic interactive services with a high quality of experience [21].

In the context of intelligent vehicles, natural elastic interaction refers to a system’s ability to dynamically adjust its responses based on varying human inputs and environmental conditions. This flexibility ensures that the interaction remains effective and comfortable for the user in different driving scenarios. It involves both the adaptability of the system to handle unexpected situations and its capacity to maintain performance levels without rigid pre-defined responses.

The challenges and bottlenecks in providing adaptive and elastic human–machine interaction in intelligent vehicles are as follows: first, the diversity and complexity of perceived information and the variability of human emotions make it difficult to accurately recognize the behaviors, states, and emotions of drivers and passengers. Second, the strong but hidden nature of the spatio-temporal causal relationships between user behavior sequences and interaction intentions, driven by sparsely correlated data, makes it difficult to accurately infer the psychological motivations and interaction intentions of drivers and passengers. Third, the complexity of human emotions makes it challenging to accurately quantify the recognition and pleasure brought by personalized interactions and to use this feedback to enhance interaction models. Fourth, due to the complex nature of dynamic human behaviors and the limited adaptability of current machine algorithms to fully comprehend and predict these behaviors in real time, it is difficult to naturally judge human–machine intervention control and to achieve natural and flexible collaboration at the appropriate time. Human actions can be unpredictable and vary significantly across different situations, which poses a challenge for machines to consistently interpret and respond appropriately. Moreover, achieving a seamless collaboration requires a deep integration of contextual understanding, which is still an area that needs significant improvement in AI technology.

### 4.2. Safety of Coordinating Primary and Secondary Tasks in Cabin-Driving Integration

In autonomous vehicles, the distributed nature of the task of the driver and those that are automated, as well as the increased likelihood of coordination defects, poses anticipated functional safety issues for cockpit interactions. In vehicles with less than Level 4 autonomy, drivers need to periodically take over control. The takeover process is a potentially high-risk moment [215]. The separation of the driver from autonomous driving tasks can lead to decreased situational awareness. Therefore, the intelligent cockpit must provide clear and accurate situational state information to assist the driver in understanding the current environment and operational requirements. Designing appropriate methods and content for interaction with in-cockpit occupants and external traffic participants is essential for improving the drivers’ situational awareness and ability to control after taking over the vehicle. Reducing the cognitive load caused by interactions and ensuring the safety of primary and secondary task coordination in complex traffic scenarios are important research topics and challenges for high safety in intelligent cockpits.

### 4.3. Lack of High-Value Human–Vehicle Interaction Data

The development of traditional cockpits involves the use of rule-based scenario descriptions and intention inference methods. As cockpit functions continue to be upgraded, this rule-driven approach has shown limitations in scalability and generalization. It is also challenging to discern the true needs of drivers and passengers, leading to the development and deployment of features that target pseudo-needs. Data-driven cockpit application scenarios can more accurately depict users’ real needs. However, extracting high-value scenarios from large amounts of raw vehicle operation and human–cockpit interaction data is highly challenging. Additionally, the lack of quantitative evaluation data on user experience creates a bottleneck in accurately enhancing functions. Quantifying how intelligent cockpits provide drivers and passengers with a sense of intelligence, safety, efficiency, and pleasure to precisely guide function improvements is also a significant challenge that needs to be addressed in the current field of intelligent cockpits.

## 5. The Outlook of Intelligent Cockpits

**Figure 8:**
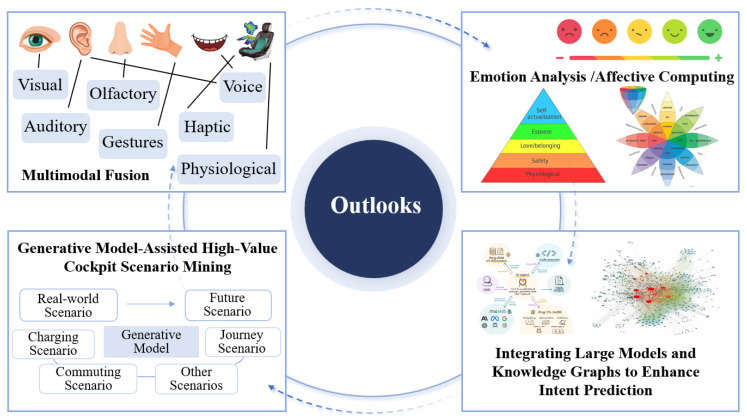
Future outlook of intelligent cockpits.

In the evolution of intelligent cockpits, significant advancements are on the horizon. By integrating multiple sensory modalities and leveraging advanced AI techniques, the cockpit environment will become more perceptive, intuitive, and responsive to occupants’ needs. The following areas highlight key trends and directions for the development of intelligent cockpits, as shown in Figure 8.

### 5.1. Enhancing Perception Performance through Multimodal Fusion

By combining multiple modes of perception, such as sound, gestures, touch, and vision, a cockpit’s perception performance can be effectively enhanced in extreme scenarios. In complex and dynamic environments, a single sensor or detection technology may encounter difficulties or false alarms. The integration of multiple sensors and technologies can allow them to complement each other, enhancing the system’s robustness and accuracy. For example, in low-light or strong-light conditions, visual sensors may fail, but sound and tactile feedback can still provide critical information to the driver. The future research could explore the optimization of sensor fusion algorithms to improve the adaptability and accuracy of multimodal systems under more varied environmental conditions.

### 5.2. Improving Understanding Capabilities through Multimodal Emotion Analysis

Emotion modeling aims to equip computers with the ability to understand and generate human emotional characteristics, enabling natural and close interactions with humans. In the future, both unimodal and multimodal emotion analysis models for cockpit occupants will continue to develop, enhancing emotional understanding capabilities and achieving natural elastic interaction for mutual adaptation between humans and vehicles. Future advancements in emotion recognition technology could leverage deeper neural networks and larger and more diverse data sets to better understand the nuances of human emotion. The research should also address the ethical considerations, privacy protection, and emotional security of occupants by developing transparent algorithms that occupants can trust and control. Moreover, the exploration of emotion adaptation models that can personalize responses based on individual preferences and historical emotional data will enhance user satisfaction and comfort.

### 5.3. Enhancing the Prediction of Interaction Intention through the Collaboration of Large Models and Knowledge Graphs

The collaboration between rules and data helps to enhance the ability to predict interaction intention. By utilizing the robust inference paradigms of large neural network models [32,216] and comprehensive knowledge coverage [217], combined with a knowledge graph of the understanding of human–cockpit intentions built from expert experience and cockpit interaction data, the accuracy of intention prediction can be significantly improved. The future research directions include the development of self-updating knowledge graphs that incorporate real-time data from the cockpit and external environment to refine predictions. Another promising area is the application of causal inference models to better understand the underlying factors driving user interactions, thereby allowing more sophisticated anticipation of user needs. Additionally, exploring federated learning approaches could enable the sharing of insights across different vehicles and models without compromising user privacy.

### 5.4. Boosting Function Satisfaction and Iteration Speed through AI and Data-Driven Cockpit Scenario Construction

High-value scenario mining and visualization technologies for big data on cockpits are the key to the development and enhancement of future data-driven cockpit functions. Data-driven approaches in the cockpit integration design allow for a precise representation of users’ actual needs. By analyzing patterns in big data from cockpit interactions, we can identify frequent and crucial usage scenarios. This targeted insight enables developers to craft cockpit functionalities that are highly responsive to user needs, ensuring both effective product development and meaningful iterations. This approach not only aligns with user expectations but also optimizes resource allocation in development processes. Furthermore, generative models [32,218] are capable of creating rich and diverse cockpit interaction scenarios, which are crucial for training deep neural networks. These models simulate scenarios that may occur infrequently in the real world but have significant implications for system performance, effectively addressing the shortcomings of the traditional dataset in scenario diversity and volume [219]. Enhanced datasets enable neural networks to learn and adapt under a broader range of conditions, thereby significantly improving the precision and efficiency of cockpit interactions. In this way, we not only enhance the ability of AI systems to handle complexity but also ensure stability and reliability across various operational environments.

The future research on intelligent cockpit systems should prioritize deep data mining and behavioral pattern recognition, continuous user experience optimization, and advancements in AI and machine learning algorithms. By enhancing techniques for analyzing big data from cockpit interactions, we can more accurately identify user behaviors and needs, allowing for the development of tailored functionalities that align closely with user expectations. This involves refining product features through iterative updates based on ongoing data analysis and feedback loops and ensuring that resources are allocated efficiently to improve user satisfaction and system performance. Additionally, researching and developing advanced AI algorithms, including deep learning and reinforcement learning, will enable cockpit systems to handle complex scenarios more effectively, fostering smarter and more adaptive capabilities. These focused areas of research will not only drive technological advancement in cockpit systems but also enhance safety, efficiency, and the overall driving experience.

## 6. Conclusions

As vehicle automation and intelligence levels increase, vehicles are gradually evolving from singular transportation tools to interconnected intelligent systems. Serving as the bridge between humans and intelligent vehicles, the form, functions, and interaction methods of intelligent cockpits are transforming, shifting from the traditional paradigm in which the human adapts to the vehicle to one in which the vehicle adapts to the human and leading toward mutual adaptation between humans and vehicles through natural interactive services. Based on the current development status of intelligent cockpits, this study first outlined the definition, intelligence levels, and technical frameworks of intelligent cockpits. Next, it proposed an interaction process for intelligent cockpits that includes multimodal perception, cognitive decision making, proactive interaction, and evolutionary evaluation. Additionally, it summarizes the key technologies of human–vehicle interaction in intelligent cockpits from the perspectives of sensing and directing interactions. Finally, it analyzes the problems and challenges faced in the field of intelligent cockpits and provides an outlook on the future development trends of human–vehicle interaction technologies in intelligent cockpits.

The progression towards highly integrated intelligent cockpits highlights a transformative approach to how vehicles interact and adapt to both their occupants and surrounding environments. As this study has delineated, the future trajectory of intelligent cockpit development will increasingly rely on sophisticated multimodal sensing and AI-driven interactions that seamlessly blend with cognitive and affective human factors. The implications of such advancements are profound, suggesting a paradigm shift where the cockpit not only enhances the driving experience but also becomes a critical component in advancing autonomous driving technologies. By refining the interaction processes through continuous learning and adaptive feedback mechanisms, future cockpits can achieve unprecedented levels of personalization and responsiveness. These advancements, however, bring forward significant challenges in ensuring data security, maintaining user privacy, and achieving system reliability, all of which must be meticulously addressed to fulfill the promise of truly intelligent cockpits that ensure safety, efficiency, and user satisfaction. The pursuit of these goals calls for a multidisciplinary approach, integrating insights from data science, human-factors engineering, and cybersecurity to pave the way for next-generation intelligent transportation systems.

## Figures and Tables

**Table 1:** Taxonomy description of intelligent cockpits.

Level	Main Features	Human–Machine Interaction	Scenario Expansion	Connected Services	Expected Date
Level 0 Traditional Cockpit	The tasks are executed in the in-cockpit scenarios; the cockpit passively responds to driver and passenger needs; it includes in-vehicle service capabilities.	Passive Interaction	Partial In-Cockpit Scenarios	In-Vehicle Infotainment System	–
Level 1 Assisted Intelligent Cockpit	The tasks are executed in the in-cockpit scenarios; the cockpit actively perceives the driver and occupants in some scenarios; the execution of the tasks requires the authorization of drivers; it includes drivers and passenger-oriented cabin service capabilities.	Cockpit Authorized Interaction	Partial In-Cockpit Scenarios	Cabin Services	2021
Level 2 Partially Cognitive Intelligent Cockpit	The tasks are executed in the partial in-cockpit and out-cockpit scenarios; the cockpit actively perceives the driver and occupants in some scenarios; the execution of the tasks is partially active; it includes open connected cloud services capabilities.	Partially Active Interaction	Partial In-Cockpit and Out-Cockpit Scenarios	Open Connected Cloud Services	2022 to 2026
Level 3 Highly Cognitive Intelligent Cockpit	The tasks are executed in the full in-cockpit and partial out-cockpit scenarios; the cockpit actively perceives the driver and occupants in the full in-cockpit scenarios; some of the tasks are executed actively; it includes cloud control platform services capabilities.	Partially Active Interaction	All In-Cockpit and Partial Out-Cockpit Scenarios	Cloud Control Platform Services	2027 to 2031
Level 4 Fully Cognitive Intelligent Cockpit	The tasks are executed across all scenarios both in-cockpit and out-cockpit; the cockpit actively perceives the driver and occupants in all in-cockpit scenarios; all of the tasks can be executed actively; it includes societal-level service capabilities.	Fully Active Interaction	All In-Cockpit and Out-Cockpit Scenarios	Societal-Level Services	2032–

**Table 3:** Directing interaction methods.

Category	Effectiveness	Works	Main Contents
Voice-based Interaction	Voice Assistant	[9]	BMW’s intelligent personal assistant.
		[154]	Mercedes-Benz’s MBUX voice assistant combined with ChatGPT.
		[155,156,157,158,159,160]	Voice assistants aid in navigation, entertainment, communication, and V2V.
	Alertness	[161]	Engaging in conversation improves driver alertness more effectively.
		[162]	Validated with the Wizard-of-Oz method that brief intermittent VA conversations improve alertness more effectively.
		[163]	Through simulated experiments, it was demonstrated that a VA with a confident tone of voice better captures the driver’s attention.
		[164]	Female voices are seen as more trustworthy, acceptable, and pleasant.
	Takeover Efficiency	[165]	Using a driving simulator, the impact of voice interfaces on driver takeover response time was evaluated.
	External Sound Recognition	[166]	A pitch detection algorithm based on signal processing aims to identify emergency vehicle sirens in real time.
		[167]	Detect emergency vehicle sirens using convolutional neural networks.
Display-based Interaction	HUD and HMD Display	[168,169]	Use stereo cameras to capture forward images and detect vehicles and pedestrians in real time with SVM.
		[170]	Achieving registration requires recognizing the posture of vehicles, heads, and eyes, as well as external targets.
		[171]	Establish a real-time registration method for 3D tracking of virtual images with the environment using feature image information.
		[172]	Improve depth perception accuracy by hiding HUD images during vehicle vibrations and utilizing human visual continuity.
		[173]	Reviewed AR-system-based registration methods comprehensively.
		[174]	Adaptively adjust AR HUD brightness and color based on ambient light.
	3D Display	[175]	Explore the application of autostereoscopic 3D vehicle dashboards, where quasi-3D displays show better performance.
	Rearview Mirror	[176]	Developed a user-centered, camera-based interior rearview mirror that switches between a traditional glass mirror and a display screen.
		[177]	Proposed a rear-stitched view panorama system using four rear cameras.
	A-Pillar	[178,179,180]	Improve A-pillar visibility with simple, regular cutouts.
		[86]	Project real-time images onto the A-pillar to show the driver the external environment blocked by the A-pillar.
Haptic-based Interaction	Haptic Descriptor	[181,182,183]	Use haptic descriptors to assist machine learning in classification.
		[182,184]	Use haptic descriptors and machine learning to classify actions and gestures.
		[185,186]	Utilize methods such as active learning and active recognition to improve the accuracy of object recognition using haptic information.
	Sensor Perception	[87]	Adopt a new active armrest with capacitive proximity sensors to achieve touch and gesture interaction and recognition.
	Haptic Warning Feedback	[187]	Provide haptic assistance during reverse training using steering wheel torque to prevent collisions during parking.
		[188]	Evaluate haptic feedback on lane departure using a driving simulator.
		[189]	Assess the impact of haptic feedback on the accelerator pedal on speeding behavior through speed records and driver interviews.
	Haptic Information Feedback	[88]	Use a wearable belt with eight haptic devices for navigation assistance.
		[190]	Embed a 5 × 5 haptic matrix in the seat for navigation directions.
Multimodal Directing Interaction	Voice + Visual + Haptic	[191]	Combine voice with visual or haptic feedback to establish a more natural interactive dialogue system.
	Voice + Gesture	[192]	Combine voice and gestures to control in-vehicle functions.
	Voice + Visual	[193]	Enhance voice interactions by combining voice and visual feedback with visualized dialogues.
	Haptic + Voice	[194]	Enhance VUI with multi-touch input and high-resolution haptic output.
	Gesture + Visual	[195]	Combine gestures with visual modes (such as HUD) to control menus.

## Data Availability

The data presented in this study are available upon request from the corresponding author.
